# Bovine Abortions Revisited—Enhancing Abortion Diagnostics by 16S rDNA Amplicon Sequencing and Fluorescence *in situ* Hybridization

**DOI:** 10.3389/fvets.2021.623666

**Published:** 2021-02-23

**Authors:** Godelind Alma Wolf-Jäckel, Mikael Lenz Strube, Kirstine Klitgaard Schou, Christiane Schnee, Jørgen S. Agerholm, Tim Kåre Jensen

**Affiliations:** ^1^National Veterinary Institute, Technical University of Denmark, Kongens Lyngby, Denmark; ^2^Department of Biotechnology and Biomedicine, Technical University of Denmark, Kongens Lyngby, Denmark; ^3^Institute of Molecular Pathogenesis, Friedrich-Loeffler-Institut, Federal Research Institute for Animal Health, Jena, Germany; ^4^Section for Veterinary Reproduction and Obstetrics, Department of Veterinary Clinical Sciences, Faculty of Health and Medical Sciences, University of Copenhagen, Taastrup, Denmark

**Keywords:** *Chlamydiaceae*, culture-independent, chlamydia-like organisms (CLO), deep sequencing, diagnostics, fluorescence *in situ* hybridization (FISH), lesion association, zoonosis

## Abstract

Abortion in cattle causes significant economic losses for cattle farmers worldwide. The diversity of abortifacients makes abortion diagnostics a complex and challenging discipline that additionally is restrained by time and economy. Microbial culture has traditionally been an important method for the identification of bacterial and mycotic abortifacients. However, it comes with the inherent bias of favoring the easy-to-culture species, e.g., those that do not require cell culture, pre-enrichment, a variety of selective growth media, or different oxygen levels for *in vitro* growth. Molecular methods such as polymerase chain reaction (PCR) and next-generation sequencing have been established as alternatives to traditional microbial culturing methods in several diagnostic fields including abortion diagnostics. Fluorescence *in situ* hybridization (FISH), a bridging microscopy technique that combines molecular accuracy with culture independence, and spatial resolution of the pathogen-lesion relation, is also gaining influence in several diagnostic fields. In this study, real-time quantitative PCR (qPCR), 16S rDNA amplicon sequencing, and FISH were applied separately and in combination in order to (i) identify potentially abortifacient bacteria without the bias of culturability, (ii) increase the diagnostic rate using combined molecular methods, (iii) investigate the presence of the difficult-to-culture zoonotic agents *Coxiella burnetii, Chlamydia* spp., and *Leptospira* spp. in bovine abortions in Denmark. Tissues from 162 aborted or stillborn bovine fetuses and placentas submitted for routine diagnostics were screened for pathogenic bacteria using 16S rDNA amplicon sequencing. Lesion association of fungal elements, as well as of selection of bacterial abortifacients, was assessed using specific FISH assays. The presence of *Chlamydia* spp. and chlamydia-like organisms was assessed using qPCR. The study focused on bacterial and fungal abortifacients, because Danish cattle is free from most viral abortifacients. The 16S rDNA amplicon sequencing–guided FISH approach was suitable for enhancing abortion diagnostics, i.e., the diagnostic rate for cases with tissue lesions (*n* = 115) was increased from 46 to 53% when compared to routine diagnostic methods. Identification of *Bacillus licheniformis, Escherichia coli*, and *Trueperella pyogenes* accounted for the majority of additional cases with an established etiology. No evidence for emerging or epizootic bacterial pathogens was found. The difficult-to-culture abortifacients were either not detected or not identified as abortifacients.

## Introduction

Abortion in cattle causes significant economic losses for farmers worldwide. Bovine abortion diagnostics is a complex, expensive, and time-consuming field, which *inter alia* is due to the variety of abortifacients including bacteria, protozoa, viruses, and fungi.

Microbial culture continues to be an important diagnostic tool in bovine abortion diagnostics (1, 2). However, because of the time-consuming nature of the method, its costliness, and inherent culturability bias, molecular, culture-independent methods are gaining importance for the detection and identification of pathogens in veterinary diagnostics including bovine abortion diagnostics (3, 4). DNA and RNA recovery methods, such as polymerase chain reaction (PCR) and next-generation sequencing, come with the advantages of detecting pathogens based on their nucleic acid sequences and thereby allowing for an efficient, highly sensitive, and specific screening for a variety of pathogens including emerging, opportunistic, and “difficult-to-culture” species (5, 6).

In bovine abortion diagnostics, the establishment of a causal relationship between the detected abortifacient and placental or fetal lesions is crucial for making an etiologic diagnosis, especially when facultative abortigenic pathogens such as *Coxiella burnetii* and ubiquitous pathogens such as *Escherichia coli* are involved (7). Moreover, abortion material is often considered as compromised because of autolysis and putrefaction, which further underlines the importance of investigating the pathogen–lesion association. Fluorescence *in situ* hybridization (FISH) is a bridging microscopy technique that combines molecular accuracy with microbial culture-independence and spatial resolution of the pathogen location within the tissue. It is therefore well-suited to evaluate the pathogen–lesion association and helps to enhance diagnoses (8–10). FISH visualizes microbial cells by fluorescently labeled nucleotide probes that bind complementarily to the ribosomal RNA (rRNA) of the target cell, a molecule present in large numbers in cells with active or just recently terminated protein expression. The rRNA's composition of variable and conserved regions makes it possible to find short sequence stretches that are unique to a species, genus, or a broader taxonomic unit, thereby allowing for different levels of differentiation between pathogenic taxa, as well as species identification in samples containing multiple species (8). FISH has lately been applied successfully in detecting and identifying *C. burnetii, Campylobacter* spp., and *Fusobacterium necrophorum* in ruminant abortions (10–12).

Another crucial aspect of bovine abortion diagnostics is the detection and identification of the difficult-to-culture and zoonotic bacteria *Chlamydia* spp., *C. burnetii*, and *Leptospira* spp. PCR, sequencing, and FISH have lately been applied individually for the detection of these agents in bovine abortions (11, 13, 14). Denmark is in the fortunate position of being free from many important abortigenic pathogens, especially viruses. For example, bovine herpesvirus type 1 has been eradicated, and eradication of bovine viral diarrhea virus (BVDV) is almost complete (15). Further, *Campylobacter fetus* subsp. *venerealis* and *Tritrichomonas foetus* are not present anymore, and bovine brucellosis was eradicated with the last case diagnosed in 1962. *Chlamydia* spp. and *Leptospira* spp. have never been diagnosed as cause of bovine abortion in Denmark; however, the routine methods included in the national surveillance program neither specifically target these agents nor *C. burnetii*, and the current prevalence of these infections is therefore uncertain.

The diagnostic rate of most bovine abortion studies is generally unsatisfyingly low and seldom reaches 50% (7, 16–19). Applying molecular high-throughput and *in situ* detection methods could enhance the diagnostic rate, e.g., by detecting opportunistic pathogens that might be missed by conventional methods.

Here, we evaluate the diagnostic benefit of applying real-time quantitative PCR (qPCR), 16S rDNA amplicon sequencing, and FISH separately and in combination in bovine abortion diagnostics. The aims of the study were to (i) identify potentially abortifacient bacteria without the bias of culturability, (ii) increase the diagnostic rate using combined molecular methods, (iii) investigate the presence of the difficult-to-culture zoonotic agents *C. burnetii, Chlamydia* spp., and *Leptospira* spp. in bovine abortions in Denmark.

## Materials and Methods

Molecular, culture-independent methods were applied to abortion material from 162 abortion cases from a previous diagnostic study that mainly applied routine diagnostic methods such as necropsy, bacterial culture, and histopathology (20). Additional tissue samples were collected and stored at −80°C for the present study. Information about the animals, reproduction data, and necropsy, histopathology, and bacterial culture findings as well the diagnostic criteria used in the routine diagnostic study are summarized in the Supplementary File 1.

### Extraction of Nucleic Acids

DNA was extracted from placenta, fetal lung, liver, and kidney. Kidney was included in order to screen for the presence of *Leptospira* spp. DNA by sequencing and PCR. Tissue samples were thawed, and 20 mg of each tissue was transferred to a sterile plastic tube using a sterile scalpel and sterile forceps. Lung and liver samples were pooled by transferring 10 mg of each tissue into one tube. If only lung or only liver was available, 20 mg of the respective tissue was used. Samples were incubated at 37°C for 30 min in 300 mL lysozyme buffer (20 mM Tris-HCl, 2 mM EDTA, 1.2% Triton X and 5 mg lysozyme per 100 mL) and 350 mL of lysis buffer (Promega, Madison, WI, USA), and a sterile 5-mm steel bead (Qiagen, Hilden, Germany) was added, and the tissue was homogenized using a TissueLyser II (Qiagen) at 20 Hz for 2 min. Protease K was added, and the sample was incubated at 56°C for 1 h. DNA was extracted on a Maxwell 16 Research Instrument System (Promega), using a Maxwell LEV Blood DNA Purification Kit (Promega). In order to control for potential contamination with 16S rDNA possibly originating from reagents or instruments, an extraction control was added for every 15 samples; i.e., a sterile plastic tube without tissue was processed in the same way and with the same reagents as the 15 samples.

### Preparation of 16S rDNA Amplicon Libraries and Sequencing

The V1–V2 region of the16 S rRNA gene was amplified using the forward primer mix 27f-YM+3, which consisted of four parts 27f-YM 5′-AGA GTT TGA TYM TGG CTC AG-3′ plus one part each of 27f-Bif 5′-AGG GTT CGA TTC TGG CTC AG-3′, 27f-Bor 5′-AGA GTT TGA TCC TGG CTT AG-3′, and 27f-Chl 5′-AGA ATT TGA TCT TGG TTC AG-3′ (21). V1V2rev 5′-CTG CTG CCT YCC GTA-3′ was used as reverse primer (22). All primers were tagged with unique nonameric (= conisting of nine subunits) barcodes to allow for multiplexing of samples (23). The predicted amplicon length was 380 base pairs (bp). The reaction was carried out in 50-μL reaction mixtures containing 5 μL of AmpliTaq Gold buffer 10x (Applied Biosystems, Branchburg, NJ, USA), 2 μL of mixed forward and reverse primer (20 μM), 1 μL of 10 mM deoxynucleoside triphosphates, 3 μL of 25 mM MgCl_2_, 0.5 μL of AmpliTaq Gold polymerase (5 U/μL; Applied Biosystems), 37.5 μL of nuclease-free water, and 1 μL of DNA template. Cycling conditions were 94°C for 6 min; 35 cycles of 94°C for 45s, 57°C for 45 s, and 72°C for 90 s followed by a final elongation at 72°C for 10 min. The samples' respective extraction controls and one no-template control (NTC) were included in each PCR run. Purified DNA from *C. burnetii, Chlamydia abortus, Chlamydia pecorum, Chlamydia psittaci*, and *Leptospira interrogans* was amplified in the same way and was used as positive control: each species separately and additionally all five species together as one mixed positive control. DNA concentration and purity of the resulting PCR products were analyzed on an Agilent 2100 Bioanalyzer using the Agilent DNA 1000 kit (Agilent Technologies, Waldbronn, Germany). PCR products were pooled in equimolar ratios of 50 ng per sample. For the controls, all available PCR product up to 50 ng was used. Each pool consisted of a total of 49 samples and their respective controls. The pooled DNA was purified using the QiagenMinElute PCR purification kit (Qiagen) according to the manufacturer's instructions. The DNA pools were sequenced on a HiSeq 250PE platform (Illumina, San Diego, CA, USA) at the National High-Throughput DNA Sequencing Centre, University of Copenhagen, Denmark.

### Analysis of 16S rDNA Amplicon Sequencing Data

The V1–V2 amplicons were merged, quality filtered, chimera-checked, and mapped against the RDP-II SSU database (http://rdp.cme.msu.edu) using the BION-meta software (Danish Genome Institute, Aarhus, Denmark). Demultiplexing was performed according to the primer and barcode sequences. Forward and reverse sequences were joined, allowing no gaps and requiring a minimum similarity of 85% as well as a sequence overlap of minimum 20 bp. Paired sequences were trimmed with a 99% quality minimum in a 20-bp window; one mutation was allowed in the primers, and the minimum length of each sequence was set to 300 bp after joining. The sequences were dereplicated, i.e., similar sequences unified, and reads with a chimera score greater than 25 were removed. The consensus sequences at 97% identity were mapped against the RDP database with a match minimum of 60%, and the taxonomical classification was based on the best 1% of the similarities from the RDP database. All bacterial 16S rDNA amplicon sequence reads present in the extraction controls and NTCs were computationally removed from the sequences of their respective samples. All samples were normalized to 100,000 reads before further analysis. Sequences were deposited as a bioproject in the NCBI sequence read archive with the accession number PRJNA678972 (www.ncbi.nlm.nih.gov/bioproject/PRJNA678972).

### *Chlamydiaceae* 23S qPCR

Based on the *Chlamydiales*-negative sequencing results for the mixed positive controls containing *Chlamydia* DNA as well as *C. burnetii* and *L. interrogans* DNA., an amplification bias of our sequencing assay against *Chlamydia* spp. and chlamydia-like organisms (CLOs) was presumed. Therefore, specific qPCR assays for the detection of *Chlamydiaceae* and *Chlamydiales* DNA were applied additionally.

DNA extracts (*n* = 162) from 127 placentas, 34 lung–liver pools, and 1 lung (latter two from cases in which DNA from placenta was not available) were screened for *Chlamydiaceae* DNA using the OIE reference method, a specific qPCR targeting a 23S rRNA gene fragment, as described previously (24). In order to distinguish true target negatives from PCR inhibition, an internal amplification control was integrated in duplex PCR runs; i.e., 500 copies of a plasmid template (Intype IC-DNA; Indical Bioscience, Leipzig, Germany) together with the corresponding primers and probe were added to each reaction as described previously (25). The presence of amplifiable sample DNA was verified using a qPCR assay targeting bovine β-actin as described previously (26). All primer and probe sets are summarized in Supplementary Table 1. Sample DNA was diluted 10-fold, and 2 μL of diluted DNA was used per 15-μL reaction. All analyses were run on a CFX96 Touch™ real-time PCR detection system (Bio-Rad, Munich, Germany). The following thermal profile was used: initial denaturation at 95°C for 10 min, 45 cycles of 95°C for 15 s, and 60°C for 60 s. For the *Chlamydiaceae* assay, an analytical cutoff value of cycle threshold (Ct) 38.0 was selected corresponding to the defined lower detection limit of the assay. Samples with a *Chlamydiaceae* Ct value ≤ 38.0 were considered positive. Samples with a Ct value >38.0 were considered negative. DNA from the cell culture–derived bovine *C. psittaci* strain DC15 was used as positive control and DNase- and RNase-free water (Qiagen) as negative control. Positive and negative controls were run with each assay. The samples and controls were analyzed in duplicate.

### Pan-*Chlamydiales* 16S qPCR

DNA extracts from the 162 abortion cases consisting of 127 placentas, 44 lung–liver pools, and 1 lung were screened for the presence of *Chlamydiales* DNA using a qPCR assay targeting the 16S rRNA gene as described previously (27). The primers and probe are listed in Supplementary Table 1. Per 15-μL reaction mixture, 2 μL of 10-fold diluted sample DNA was used. All samples were positive for the presence of amplifiable DNA when analyzed in the *Chlamydiaceae* 23S assay; therefore, no additional amplification control was used in this assay. All analyses were run on a CFX96 Touch™ real-time PCR detection system (Bio-Rad). The following thermal profile was used: initial denaturation at 95°C for 10 min, 45 cycles of 95°C for 15 s, 67°C for 30 s, and 72°C for 30 s. Samples with an average Ct value ≤ 38.0 were considered positive, whereas samples with an average Ct value >38.0 were considered negative. DNA from the cell culture–derived bovine *C. psittaci* strain DC15 was used as positive control and DNase- and RNase-free water (Qiagen) as negative control. Positive and negative controls were run with each assay. The samples and controls were analyzed in duplicate.

### *Clamydiales* 16S Sequencing

For all *Chlamydiales* PCR-positive samples (*n* = 9), the *Chlamydiales* PCR was repeated in a larger reaction mixture volume of 50 μL in order to generate enough amplicon DNA for sequencing. The PCR product (expected length ca. 210 bp) was then purified and extracted using the FastGene PCR Extraction kit (Nippon Genetics, Düren, Germany) according to the manufacturer's instructions. The primers panCh16F2 and panCh16R2 (Supplementary Table 1) were added to the purified DNA, and the samples were submitted to Sanger sequencing (Eurofins Genomics, Ebersberg, Germany). The obtained sequences were edited manually and subjected to a BLASTn search of the 16S rRNA (bacteria- and Archaea-type strains) database (https://blast.ncbi.nlm.nih.gov).

### *Leptospira* spp. qPCR

Two previously described qPCR assays for the specific detection of pathogenic *Leptospira* spp. were used in order to further investigate the abortion case from which the only *L. interrogans* 16S rDNA amplicon sequencing–positive sample (liver) originated (28, 29). DNA samples from kidney, liver, and placenta of this case were tested in duplicate with both assays. One assay targeted the 16S rRNA gene (28); the other, the *lipL32* gene of pathogenic *Leptospira* spp. (29).

### FISH Screening for Selected Bacterial Species and Fungi

The abortion cases with microscopic placental and/or fetal lesions suggestive of infection (diagnostic groups 1 and 2 according to Supplementary File 1) were examined for lesion association of fungi and selected bacterial species using FISH according to the inclusion criteria listed in Table 1.

**Table 1:** Results of the sequencing-based FISH analysis of selected abortion cases from routine diagnostic groups 1 and 2 (20).

**Species**	**FISH screening inclusion criteria**	**No. of cases screened**	**No. of cases of agent-associated lesions**	**Previous etiology (routine methods)^**a**^**	**Final etiology (combined methods)^**b**^**
*B. licheniformis*	>10,000 reads^c^ placenta and/or culture positive ≥1 organ	4	4	*B. licheniformis n* = 1 Unknown *n* = 3	*B. licheniformis n* = 3 *B. licheniformis* + *E. coli n* = 1
*C. burnetii*	all cases with *C. burnetii* reads^c^	3	0	*T. pyogenes n* = 1 *K. pneumoniae n* = 1 Unknown *n* = 1	*T. pyogenes n* = 1 *K. pneumoniae n* = 1 Unknown *n* = 1
*C. jejuni*	all cases with *C. jejuni* reads^c^	1	0	Unknown *n* = 1	*E. coli n* = 1
*E. coli*	>20,000 reads^c^ placenta^d^ and/or pure culture positive ≥1 organ	21	8	*E. coli n* = 4 *N. caninum n* = 6 Unknown *n* = 11	*E. coli n* = 6 *B. licheniformis* + *E. coli n* = 1 *N. caninum n* = 5 *N. caninum* + *E. coli n* = 1 Unknown *n* = 8
*F. necrophorum*	>10,000 reads^c^ placenta	7	0	*E. coli n* = 1 *N. caninum n* = 3 Unknown *n* = 3	*E. coli n* = 1 *N. caninum n* = 3 *T. pyogenes n* = 2 Unknown *n* = 1
*L. monocytogenes*	>10,000 reads^c^ placenta and/or culture positive ≥1 organ	2	2	*L. monocytogenes n* = 2	*L. monocytogenes n* = 2
*S. aureus*	>10,000 reads^c^ placenta and/or culture positive ≥1 organ	4	3	*S. aureus n* = 3 Unknown *n* = 1	*S. aureus n* = 3 Unknown *n* = 1
*T. pyogenes*	>10,000 reads^c^ placenta^d^ and/or culture positive ≥1 organ	8	8	*T. pyogenes n* = 5 BVDV *n* = 1 Unknown *n* = 2	*T. pyogenes n* = 7 *T. pyogenes* + BVDV *n* = 1

BVDV, bovine viral diarrhea virus.

a The previous etiologic diagnosis was made according to the diagnostic criteria of the routine diagnostic study (20).

b The final etiologic diagnosis was made based on a combined evaluation of the bacterial culture, histopathologic, sequencing, and FISH findings.

c 16S rDNA amplicon sequencing reads of the respective bacterial species.

d If placenta was not available, the number of sequencing reads from the lung–liver pool or lung were assessed and the cases were FISH-screened if the number of reads was above the inclusion limit for placenta.

*Species-specific probes were applied to evaluate the causal relationship between the pathogen and tissue lesions*.

Sections of fetal placenta, lung, liver, and kidney were mounted on Superfrost Plus^TM^ slides (Gerhard Menzel, Braunschweig, Germany) and hybridized as described previously (11). In brief, hybridization was carried out at 45°C (50°C for probe Fnecr) for 16 h and at a final probe concentration of 5 ng/μL. After hybridization, the slides were washed in washing buffer, rinsed in water, air dried, and mounted in Vectashield (Vector Laboratories Inc., Burlingame, CA, USA) for fluorescence microscopy. The oligonucleotide probes (Eurofins Genomics) were 5′-labeled with either fluorescein isothiocyanate (FITC), cyanine 3 (Cy3), or Alexa Fluor 555 (AF555). An Axioimager M1 microscope (Carl Zeiss, Oberkochen, Germany), equipped for epifluorescence with a 100-W HBO lamp and filter sets 24 (excitation at 485/578 nm), 38 (excitation at 470 nm), and 43 (excitation at 550 nm) for the detection of double staining (FITC and Cy3 or AF555) and single staining (FITC, Cy3, AF555), respectively, was used to examine the hybridized specimens. Images were obtained using an AxioCam MRm version 3 FireWire monochrome camera and the AxioVision software, version 4.5 (Carl Zeiss). Probe sequences, targets, systematic names, and references are listed in Table 2.

**Table 2:** FISH probes that were applied for the detection and assessment of lesion association of abortifacients in placenta and fetal organs.

**Probe ID**	**Target**	**Systematic name^**a**^**	**Sequence 5^**′**^-3^**′**^**	**References**
Blich	*B. licheniformis*	S-S-Blich-0079-a-A-21	Cy3-CTGACCTAAGGGAGCAAGCTC	(30)^b^
Cajej	*C. jejuni*	L-S-Cajej-1693-a-A-21	Cy3-AGCTAACCACACCTTATACCG	(31)
Cburn	*C. burnetii*	S-S-Cburn-0443-a-A-18	AF555-CTTGAGAATTTCTTCCCC	(11)
D223	Fungi	L-D-Fungi-0223-a-A-18	Cy3-CCACCCACTTAGAGCTGC	(32)
Eco45a	*E. coli*	L-S-Ecoli-1161-a-A-18	Cy3-gcataagcgtcgctgccg	(33)
EUB338	Domain bacteria	S-D-Bact-0338-a-A-18	FITC-GCTGCCTCCCGTAGGAGT	(34)
Fnecr	*F. necrophorum*	S-S-Fnecr-0183-a-A-18	Cy3-gattcctccatgcgaaaa	(12)
Lm-16S-2	*L. monocytogenes*	S-S-Lmono-1243-a-A-18	Cy3-CGACCCTTTGTACTA	(35)
Sau	*S. aureus*	S-S-Saure-0069-a-A-19	Cy3-GAAGCAAGCTTCTCGTCCG	(36)
Tpyo	*T. pyogenes*	S-S-Apyog-0464-a-A-18	Cy3-gcacataccgtcacaaaa	(37)

a According to The Oligonucleotide Probe Database nomenclature (38).

b *The reverse complement sequence of the B. licheniformis–specific TaqMan probe was used*.

For the evaluation of the amount and localization of bacterial cells and microcolonies, the general bacterial probe EUB338 was applied. Screening for the presence of fungal elements was performed on the placenta and/or lung from all abortion cases using the pan fungal probe D223. In order to screen for a selected panel of bacterial species, specific Cy3-labeled probes were used either alone or as double hybridization with the FITC-labeled EUB338 probe. The species were chosen based on the abortifacients known to cause bovine abortion in Denmark (17, 20), as well as on the availability of evaluated FISH probes.

FISH controls were prepared by injecting pure bacterial cultures suspended in a 0.9% sterile saline solution into sterile porcine lung samples. The tissue was then fixed in 10% neutral-buffered formalin, trimmed, processed routinely, embedded in paraffin, and cut into 3–5 μm sections. The bacterial species and strains are listed in Supplementary Table 2. Tissue sections from cases of experimental and spontaneous infections with *Bacillus licheniformis, C. abortus, C. pecorum, Listeria monocytogenes, C. burnetii, E. coli*, and a fungus were used as further FISH controls (Supplementary Table 2).

Probe Blich was derived from a previously published *B. licheniformis* specific Taqman qPCR probe (30) by using the reverse complement sequence and adding Cy3 at the 5′-end. The sensitivity and specificity of probe Blich were evaluated with the *B. licheniformis*–positive control (positive FISH signal), as well as the *L. monocytogenes*– and *E. coli*–positive controls (no FISH signal).

Bacteria were regarded as lesion-associated when a specific fluorescence signal was detected in association with tissue lesions. Lesions were recognized either based on the tissue structure visible during fluorescence microscopy or by identifying and evaluating the corresponding region of interest on a serial hematoxylin-eosin–stained tissue section. Bacterial cells and microcolonies were in general considered lesion-associated when they were found in the immediate proximity of tissue lesions, e.g., in areas with inflammation and/or necrosis. In the placenta, presence in areas of trophoblast swelling, sloughing, and necrosis; attachment to necrotic chorionic villi; and intracellular localization in trophoblasts and phagocytes were considered lesion-associated. In the fetal lung, bacterial localization within cellular debris in the airways and intracellularly in phagocytes was considered lesion-associated.

### Statistical Test

A χ^2^ test for equal proportions was used to test if the proportion of diagnosed cases was different between the routine and combined approaches.

## Results

### 16S rDNA Amplicon Sequencing

After demultiplexing, sequence cleaning, uniquification, and chimera filtering of the 16S rDNA amplicon sequences obtained from all samples and controls, 92,999,372 sequences were available for taxonomic classification, whereof 86,773,730 (93%) were mapped to a unique bacterial species. The resulting data set was diminished by potential contaminant sequences shared with NTCs and extraction controls and was then analyzed focusing on the following aspects.

#### Difficult-to-Culture Bacterial Abortifacients

Three cases were sequencing-positive for *C. burnetii* (Table 1, Supplementary Table 4). The abundance of *C. burnetii* was generally low and ranged from 0 to 1,085 reads in the placenta samples. One case was sequencing-positive for *Leptospira* spp. with a single *L. interrogans* read detected in the liver. Sequences of the *Chlamydiaceae* family and CLOs were not detected in any case. The positive controls for *C. burnetii, Chlamydia* spp., and *L. interrogans* were sequencing-positive when amplified separately. In the mixed positive control containing DNA from all five species, only *C. burnetii* and *L. interrogans* reads were detected.

#### Potentially Epizootic and/or Emerging Bacterial Abortifacients

The most prevalent bacteria with the highest abundance were *E. coli* (12 cases), *Streptococcus pluranimalium* (11 cases), and *S. equinus* (eight cases) in placenta (Table 3); *Psychrobacter psychrophilus* (13 cases), *Acinetobacter* sp. (12 cases), and *Aerococcus viridans* (11 cases) in lung and/or liver (Table 4); and *Acinetobacter* sp. (11 cases), *Aerococcus viridans* (10 cases), *Facklamia* sp., and *Psychropbacter psychrophilus* (10 cases each) in kidney (Table 5).

**Table 3:** Prevalence of the most abundant bacterial taxa in placenta from bovine abortion cases based on 16S rDNA amplicon sequencing.

**Most abundant taxa placenta**	**No. of abortion cases**
*Escherichia coli*	12
*Streptococcus pluranimalium*	11
*Streptococcus equinus*	8
*Aerococcus viridans*	6
*Clostridium perfringens*	6
*Facklamia* sp.	5
*Fusobacterium necrophorum*	5
*Psychrobacter psychrophilus*	5
*Clostridium bifermentans*	4
*Mannheimia varigena*	4
*Fusobacterium periodonticum*	3
*Fusobacterium varium*	3
*Staphylococcus aureus*	3
*Trueperella pyogenes*	3
*Acinetobacter johnsonii*	2
*Bacillus licheniformis*	2
*Bacteroides* sp.	2
*Clostridium* sp.	2
*Enterococcus faecalis*	2
*Lactococcus garvieae*	2
*Listeria monocytogenes*	2
*Peptostreptococcus russellii*	2
*Staphylococcus vitulinus*	2
*Streptococcus parauberis*	2
*Vagococcus fluvialis*	2
*Acinetobacter lwoffii*	1
*Acinetobacter* sp.	1
*Aeromonas sobria*	1
*Atopostipes* sp.	1
*Bacteroides pyogenes*	1
*Clostridium septicum*	1
*Clostridium sordellii*	1
*Corynebacterium* sp.	1
*Gemella*	1
*Hafnia alvei*	1
*Helcococcus* sp.	1
*Jeotgalicoccus* sp.	1
*Kurthia gibsonii*	1
*Lactococcus* sp.	1
*Mannheimia granulomatis*	1
*Myroides* sp.	1
*Prevotella* sp.	1
*Pseudomonas psychrophila*	1
*Pseudomonas* sp.	1
*Psychrobacter* sp.	1
*Psychrobacter* sp., *Facklamia* sp.	1
*Shigella sonnei*	1
*Staphylococcus equorum*	1
*Staphylococcus haemolyticus*	1
*Staphylococcus rostri*	1
*Streptococcus dysgalactiae*	1
*Streptococcus uberis*	1
Total	127

*Sequencing data were available for placenta in 127 of 162 cases. In twin abortions with sequencing data available from both fetuses, both most abundant taxa are listed if not identical*.

**Table 4:** Prevalence of the most abundant bacterial taxa in fetal lung and/or liver from bovine abortion cases based on 16S rDNA amplicon sequencing.

**Most abundant taxa lung and/or liver**	**No. of abortion cases**
*Psychrobacter psychrophilus*	13
*Acinetobacter* sp.	12
*Aerococcus viridans*	11
*Facklamia* sp.	7
*Lactococcus lactis*	7
*Trueperella pyogenes*	6
*Clostridium sordellii*	5
*Streptococcus pluranimalium*	5
*Acinetobacter lwoffii*	4
*Clostridium bifermentans*	4
*Lactococcus* sp.	4
*Escherichia coli*	3
*Fusobacterium necrophorum*	3
*Lysinibacillus* sp.	3
*Proteus vulgaris*	3
*Psychrobacter* sp.	3
*Staphylococcus equorum*	3
*Vibrio vulnificus*	3
*Acinetobacter baumannii*	2
*Bacilli* sp.	2
*Corynebacterium* sp.	2
*Kurthia zopfii*	2
*Listeria monocytogenes*	2
*Pseudomonas deceptionensis*	2
*Shigella sonnei*	2
*Staphylococcus aureus*	2
*Weissella hellenica*	2
*Aeromonas hydrophila*	1
*Aeromonas salmonicida*	1
*Aeromonas sobria*	1
*Bacillus licheniformis*	1
*Bacteroides fragilis*	1
*Bacteroidia* sp.	1
*Bacteroidia* sp., *Psychrobacter faecalis*	1
*Campylobacter jejuni*	1
*Carnobacteriaceae* sp.	1
*Clostridium butyricum*	1
*Clostridium perfringens*	1
*Clostridium septicum*	1
*Clostridium* sp., *Aeromonas hydrophila*	1
*Enterobacter hormaechei*	1
*Enterococcus avium*	1
*Enterococcus durans*	1
*Facklamia* sp., *Corynebacterium* sp.	1
*Facklamia* sp., *Psychrobacter* sp.	1
*Firmicutes* sp., *Lactococcus raffinolactis*	1
*Fusobacterium varium*	1
*Gemella*	1
*Haemophilus influenzae*	1
*Hafnia alvei*	1
*Jeotgalibaca* sp.	1
*Jeotgalicoccus* sp.	1
*Kocuria* sp.	1
*Kurthia gibsonii*	1
*Lactobacillus sakei*	1
*Lactococcus garvieae*	1
*Lactococcus lactis, Psychrobacter psychrophilus*	1
*Lactococcus raffinolactis*	1
*Lelliottia amnigena*	1
*Mannheimia varigena*	1
*Moellerella wisconsensis*	1
*Proteus mirabilis*	1
*Psychrobacter pacificensis*	1
*Romboutsia lituseburensis*	1
*Staphylococcus rostri*	1
*Staphylococcus warneri*	1
*Streptococcus equinus*	1
*Streptococcus equinus, ClostridiumXI* sp.	1
*Streptococcus parauberis*	1
*Vibrio* sp.	1
*Weissella ceti*	1
Total	161

*Sequencing data were available for lung and/or liver in 161 of 162 cases. In twin abortions with sequencing data available from both fetuses, both most abundant taxa are listed if not identical*.

**Table 5:** Prevalence of the most abundant bacterial taxa in fetal kidney from bovine abortion cases based on 16S rDNA amplicon sequencing.

**Most abundant taxa kidney**	**No. of abortion cases**
*Acinetobacter* sp.	11
*Aerococcus viridans*	10
*Facklamia* sp.	10
*Psychrobacter psychrophilus*	10
*Clostridium sordellii*	8
*Lactococcus lactis*	7
*Lysinibacillus* sp.	4
*Psychrobacter* sp.	4
*Streptococcus pluranimalium*	4
*Trueperella pyogenes*	4
*Acinetobacter lwoffii*	3
*Carnobacteriaceae* sp.	3
*Escherichia coli*	3
*Shigella sonnei*	3
*Aeromonas sobria*	2
*Bacillus licheniformis*	2
*Clostridium butyricum*	2
*Corynebacterium* sp.	2
*Fusobacterium necrophorum*	2
*Fusobacterium varium*	2
*Lactobacillus curvatus*	2
*Lactobacillus sakei*	2
*Lactococcus* sp.	2
*Listeria monocytogenes*	2
*Proteus vulgaris*	2
*Staphylococcus aureus*	2
*Staphylococcus sciuri*	2
*Streptococcus equinus*	2
*Streptococcus parauberis*	2
*Acinetobacter baumannii*	1
*Acinetobacter johnsonii*	1
*Actinomyces* sp.	1
*Aeromonas salmonicida*	1
*Arthrobacter antarcticus*	1
*Bacilli* sp.	1
*Bacteroides* sp.	1
*Bacteroidia* sp., *Psychrobacter faecalis*	1
*Chishuiella* sp.	1
*Clostridium perfringens*	1
*Clostridium septicum, Clostridium* sp.	1
*Clostridium* sp., *Proteus vulgaris*	1
*Dietzia* sp., *Peptostreptococcus russellii*	1
*Enterobacter hormaechei*	1
*Escherichia fergusonii*	1
*Firmicutes* sp., *Lactococcus lactis*	1
*Fusobacterium periodonticum*	1
*Gemella*	1
*Hafnia alvei*	1
*Kurthia* sp.	1
*Kurthia zopfii*	1
*Lactococcus garvieae*	1
*Lactococcus lactis, Psychrobacter pacificensis*	1
*Lactococcus raffinolactis*	1
*Mannheimia varigena*	1
*Myroides* sp.	1
*Pasteurella* sp.	1
*Pseudoalteromonas* sp.	1
*Pseudomonas deceptionensis*	1
*Psychrobacter pacificensis*	1
*Ralstonia pickettii, Carnobacterium* sp.	1
*Ralstonia* sp.	1
*Ralstonia* sp., *Psychrobacter* sp.	1
*Shewanella putrefaciens*	1
*Staphylococcus rostri*	1
*Staphylococcus warneri*	1
*Vibrio diazotrophicus*	1
*Vibrio vulnificus*	1
*Weissella ceti*	1
*Weissella cibaria*	1
Total	154

*Sequencing data were available for kidney in 154 out of 162 cases. In twin abortions with sequencing data available from both fetuses, both most abundant taxa are listed if not identical*.

When screening the most abundant species in placenta, lung–liver pool, and kidney, neither clustering of cases positive for bacteria known to be able to cause epizootic abortion (e.g., *Brucella abortus, Leptospira* spp., *Listeria* spp., *Salmonella* spp., *Ureaplasma diversum*), nor potentially emerging bacterial species were recognized.

The following known abortifacient taxa were not detected: *Brucella* spp., *Campylobacter fetus, Mycoplasma bovis, Chlamydia* spp., CLOs, *Listeria ivanovii, Yersinia pseudotuberculosis, Histophilus somni, Anaplasma phagocytophilum, Flexispira* spp., and *Pajaroellobacter abortibovis*.

All NTCs and the majority of extraction controls were free from 16S rDNA amplicon reads of the bacterial species that were examined for lesion association using FISH. Furthermore, the controls were sequencing-negative for *Brucella* spp., *Chlamydiaceae*, CLOs, and *Leptospira* spp. The extraction controls for 5 of the 21 cases screened for *E. coli* contained *E. coli* sequence reads (cases 8, 10, 17, 18, and 24; Supplementary Table 6). In three of these cases, the *E. coli* sequences found in the placenta and/or lung and liver samples were different from the *E. coli* sequences found in the respective negative controls (cases 8, 18, and 24; Supplementary Table 6). While case 10 was sequencing-negative for *E. coli* prior to the removal of potential contaminant sequences, case 17 became sequencing-negative for *E. coli* due to the removal of potential contaminant sequences. The extraction control for one of the eight cases screened for *Trueperella pyogenes* contained *T. pyogenes* sequence reads and removal of potential contaminant sequences turned this case into *T. pyogenes* sequencing-negative (case 40; Table 6).

**Table 6:** Diagnoses and results of bacterial culture, sequencing, fluorescence *in situ* hybridization (FISH), and histology for eight bovine abortion cases that were FISH-screened for *T. pyogenes* lesion association.

**Case ID**	**Culture *T. pyogenes*** **≥1 organ**	**Sequencing >10,000*T. pyogenes*** **reads placenta^**a**^**	**Sequencing *T. pyogenes* in extraction control**	**Sequencing *T. pyogenes*** **among three most abundant placenta**	**Sequencing *T. pyogenes* among** **three most abundant lung–liver**	**Sequencing most abundant placenta**	**Sequencing most abundant** **lung–liver**	**FISH *T. pyogenes***	**FISH *T. pyogenes*** **lesion-associated**	**Histological** **findings**	**Previous** **etiology**	**Final** **etiology**
Case 6	+	+	–	+	+	*T. pyogenes*	*T. pyogenes*	+	+	Suppurative bronchopneumonia	*T. pyogenes*	*T. pyogenes*
Case 28	–	+	–	+	+	*Bacteroides* unclassified	*Lactococcus* unclassified	+	+	Necrosuppurative placentitis Suppurative bronchopneumonia	Unknown	*T. pyogenes*
Case 33	–	+	–	+	+	*T. pyogenes*	*Enterococcus durans*	+	+	Necrosuppurative placentitis Suppurative bronchopneumonia Non-suppurative encephalitis	Unknown	*T. pyogenes*
Case 40	+	–	+	NA	–	NA	*Acinetobacter* unclassified	+	+	Suppurative bronchopneumonia	*T. pyogenes*	*T. pyogenes*
Case 41	+	+	–	+	+	*T. pyogenes*	*T. pyogenes*	+	+	Suppurative placentitis Suppurative bronchopneumonia	*T. pyogenes*	*T. pyogenes*
Case 42	+	–	–	NA	–	NA	*Bacteroides fragilis*	+	+	Suppurative bronchopneumonia	*T. pyogenes*	*T. pyogenes*
Case 43	+	+	–	+	+	*Staphylococcus vitulinus*	*T. pyogenes*	+	+	Necrosuppurative placentitis Suppurative bronchopneumonia	*T. pyogenes*	*T. pyogenes*
Case 44	–	+	–	NA	+	NA	*T. pyogenes*	+	+	Suppurative bronchopneumonia BVDV antigen in tissue	BVDV	BVDV, *T. pyogenes*

–*, no/negative; +, yes/positive; BVDV, bovine viral diarrhea virus; NA, data not available.*

a *If placenta was not available, the number of reads from the lung–liver pool or lung was assessed*.

### FISH Screening for Bacterial Infections Based on 16S rDNA Amplicon Sequencing

**Figure 1:**
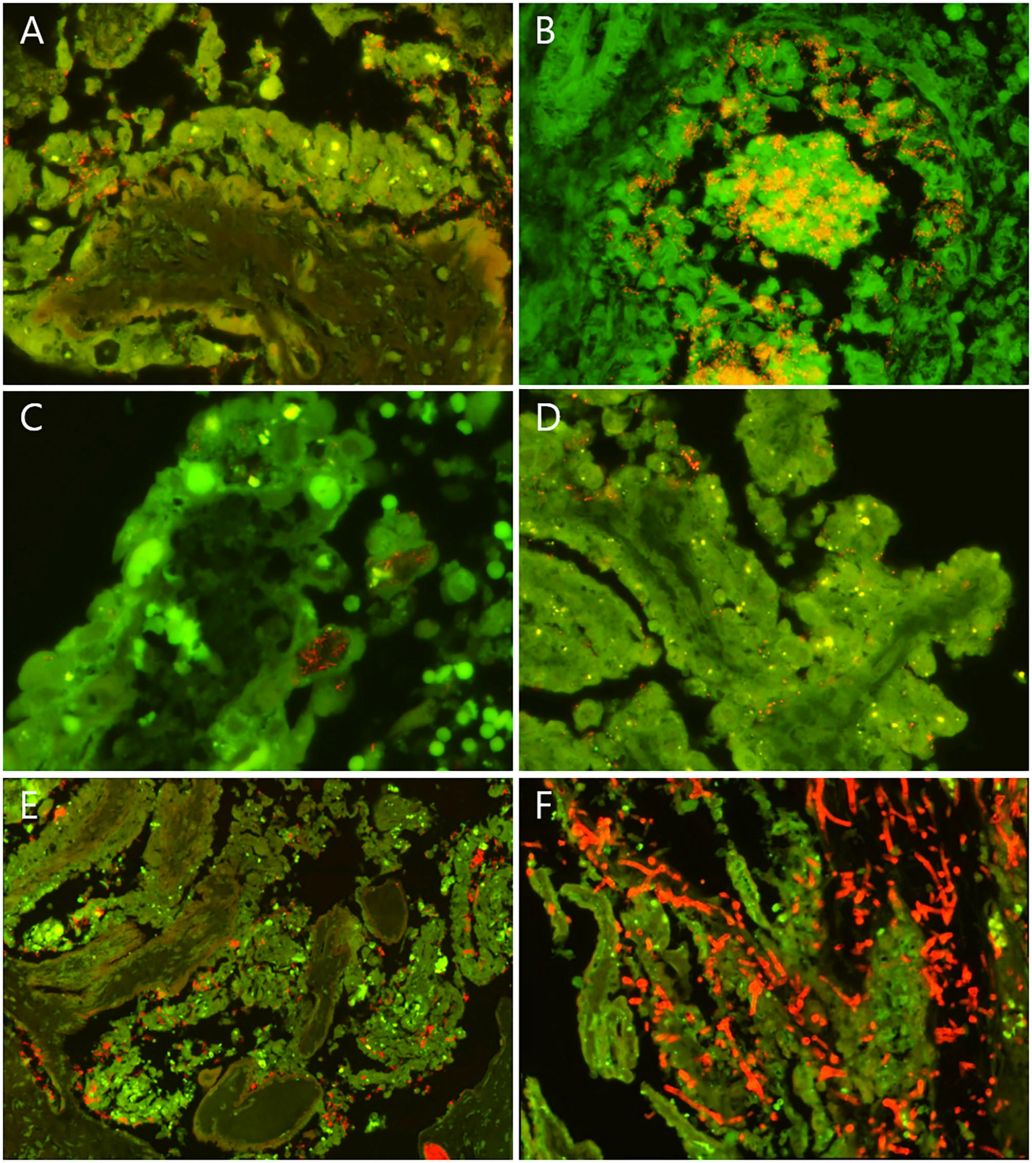
Examples of fluorescence *in situ* hybridization (FISH) findings in selected bovine abortion cases screened for lesion association of fungi and selected bacterial species. Fungi and the selected bacteria appear red/orange. Erythrocytes appear bright green. **(A)** Placenta from a case of *T. pyogenes* abortion (case 33). *T. pyogenes* cells (red/orange) colonized the cleft between the sloughing necrotic trophoblasts and the denuded basal membrane of a chorionic villus. **(B)** Fetal lung from a case of *T. pyogenes* abortion (case 41). Numerous *T. pyogenes* cells were embedded in the cellular and acellular debris in the lumen of a bronchiole and formed microcolonies. The bacteria also invaded and broke through the bronchiolar epithelium. **(C)** Tip of a chorionic villus with intracytoplasmic filamentous bacteria (red/orange) in sloughing and necrotic trophoblasts in a case of *B. licheniformis* abortion (case 4). **(D)** Placenta from a case of *S. aureus* abortion. *S. aureus* cells (red/orange) were found between rounded trophoblasts and between trophoblasts and the chorionic basal membrane. **(E)** Placenta from a case of *L. monocytogenes* infection. *L. monocytogenes* cells were associated with sloughing and necrotic trophoblasts and located in the lumen of stromal blood vessels. **(F)** Placenta from a case of mycotic abortion. Large numbers of fungal septate and branching hyphae (red/orange) infiltrated the necrotic chorionic villi. **(A–E)** FISH using the probes listed in Table 1.

A selection of 44 cases out of the 115 cases with lesions suggestive of infection (Table 7) was screened for pathogen–lesion association for a selection of bacterial species using FISH. The inclusion criteria, species, FISH results, and diagnoses are listed in Table 1. Examples of lesion association confirmed by FISH are shown in Figure 1.

**Table 7:** Comparison of diagnoses made using routine methods (20) with the diagnoses reached by combining routine methods with a 16S rDNA amplicon sequencing–guided FISH analysis.

**Diagnostic group**	**No. of cases routine methods**	**% of total routine**	**No. of cases combined methods**	**% of total combined**
***1. Likely cause of abortion identified***	53^a^	46	61^a^	53
*N. caninum*^b^	31	27	31	27
*T. pyogenes*	5	4	7	6
*E. coli*	4	4	6	5
*E. coli, B. licheniformis*	0	0	1	<1
*S. aureus*	3	3	3	3
*L. monocytogenes*	2	2	2	2
*B. licheniformis*	1	<1	3	3
Other bacterial species^c^	4	4	4	4
Fungi	2	2	3	3
BVDV	1	<1	0	0
BVDV, *T. pyogenes*	0	0	1	<1
***2. Lesions present, specific etiology not identified***	62^a^	54	54^a^	47
Suppurative to necrosuppurative placentitis and suppurative bronchopneumonia	12	10	8	7
Suppurative to necrosuppurative placentitis	26	23	21	18
Suppurative bronchopneumonia	14	12	15	13
Non-suppurative placentitis	8	7	8	7
Interstitial pneumonia	1	<1	1	<1
Granulomatous pneumonia	1	<1	1	<1
Total	115	100	115	100

BVDV, bovine viral diarrhea virus.

a Accumulated figures within the respective diagnostic group.

b In two of these cases, E. coli and L. garvieae were identified as coinfecting agents, respectively.

c Streptococcus sp., Klebsiella pneumoniae, Aeromonas sp., and Lactococcus garvieae (one case each).

*Etiologic diagnoses for 115 bovine abortion cases with lesions suggestive for infection out of 162 total cases*.

#### Bacillus licheniformis

*B. licheniformis* was found lesion-associated in the placenta of all four screened cases. The findings are summarized in Supplementary Table 3. *B. licheniformis* was among the two most abundant taxa in placenta in all four cases (Table 8). *B. licheniformis* was established as a final cause of abortion in all four cases; and in case 3 as coinfection with *E. coli*. For three of the cases, the previously established etiology was unknown.

**Table 8:** Bovine abortion cases with a final bacterial etiology (*n* = 29).

**Final etiology** **(no. of cases)**	**% of cases with final etiology among most abundant taxa**								**% of cases with final etiology not among**	
	**Placenta**			**Lung–liver pool**			**Highest abundance ≥1 site**	**Highest, 2nd or 3rd highest ≥1 site**	**Highest abundance neither site**	**Three most abundant neither site**
	**Highest**	**Highest or 2nd highest**	**Highest, 2nd or 3rd highest**	**Highest**	**Highest or 2nd highest**	**Highest, 2nd or 3rd highest**				
*S. aureus* (3)	100	100	100	67	67	67	100	100	0	0
*T. pyogenes* (8)	38^a^	38^a^	63^a^	50	50	75	63	75	38	25
*L. monocytogenes* (2)	100	100	100	100	100	100	100	100	0	0
*B. licheniformis* (4)	50	100	100	25	25	25	75	100	25	0
*Streptococcus* sp. (1)	100	100	100	0	100	100	100	100	0	0
*E. coli* (8)	63	63	63	13	25	25	63	63	38	38
*L. garvieae* (2)	50	50	50	0	50	50	50	50	50	50
*Aeromonas* sp. (1)	0	0	0	0	0	100	0	100	100	0
*K. pneumonia* (1)	0	0	0	0	0	0	0	0	100	100
Average accordance in %	56	56	64	28	46	60	61	76	NA	NA

NA, not applicable.

a Placenta was not available in three of eight cases.

*Level of accordance between the final etiology and the most sequencing abundant taxa in the placenta and/or the lung–liver pool. The final etiology was determined based on a combination of diagnostic methods (bacterial culture, histopathology, and 16S rDNA amplicon sequencing–based FISH)*.

#### Escherichia coli

*E. coli* was found lesion-associated in 8 of the 21 screened cases and was thereby established as the sole cause of abortion in six cases and as coinfecting agent together with *Neospora caninum* or *B. licheniformis*, respectively, in two cases. The findings are summarized in Supplementary Table 6. Ten of the screened cases had a previously established etiology prior to the FISH analysis: four cases were formerly diagnosed as *E. coli*–associated abortions, and six cases as *N. caninum*–associated abortions. Of the formerly *E. coli*-associated abortions, only two were confirmed by FISH, and no final etiology could be established for the other two cases. For 11 of the screened cases, the etiology was unknown prior to FISH. In five of these cases, *E. coli* was established as the final cause of abortion. Of the 12 cases, in which *E. coli* was isolated as pure culture, 10 cases were sequencing-negative for *E. coli* (including six cases with pure culture from abomasal content). *E. coli* was established as the cause of abortion in three of these cases: as sole cause in two cases and as coinfectant together with *B. licheniformis* in one case. In five cases, *E. coli* sequences were detected in the screened samples' extraction controls (Supplementary Table 6). Two of these cases were *E. coli* sequencing-negative. In one of these cases (case 10), the samples and control shared identical *E. coli* sequences, and the samples therefore became sequencing-negative for *E. coli* after removal of the potential contaminant sequences. However, based on the FISH findings, *E. coli* was nevertheless established as the final cause of abortion. The samples of the other case (case 17) did not contain any *E. coli* sequences before the removal of potential contaminant sequences. In the remaining three cases with *E. coli* sequencing-positive extraction controls, the sequences in the extraction controls and samples were not identical and therefore not removed from the data set.

#### Listeria monocytogenes

*L. monocytogenes* was found lesion-associated in both examined cases. The findings are summarized in Supplementary Table 8. In both cases, *L. monocytogenes* was the most abundant taxon in all sequenced samples and comprised between 66 and 100% of all sequencing reads per sample. *L. monocytogenes* was confirmed as the cause of abortion in both cases, thereby confirming the culture-based diagnosis.

#### Staphylococcus aureus

*S. aureus* was found lesion-associated in three of the four examined cases. The findings are summarized in Supplementary Table 9. *S. aureus* was associated with placental lesions in all three cases and with lung lesions in one of them (case 38). In the cases with lesion association, *S. aureus* was among the two most abundant taxa in the placenta with abundances ranging from 73 to 100%. The sequencing-negative case 37 was the only screened case from which *S. aureus* was isolated as mixed bacterial culture (liver), whereas a pure *S. aureus* culture was isolated from at least one organ in the three other cases. *S. aureus* was confirmed as the cause of abortion in the three cases that were identified with routine methods.

#### Trueperella pyogenes

*T. pyogenes* was detected in association with tissue lesions in all eight examined cases: associated with placental lesions in all five cases in which placenta was available and associated with lung lesions in the remaining three cases.

#### Fusobacterium necrophorum

*F. necrophorum* was not detected to be associated with potentially abortigenic lesions in any of the seven examined cases. It was found as a non-invasive part of a multispecies community associated with superficial cellular and acellular debris in the placenta. A total of 13 cases had >10,000 reads in placenta; however, six cases were excluded from the FISH screening because they belonged to diagnostic groups 3 and 4 because of the lack of tissue lesions.

*C. burnetii* and *Campylobacter jejuni* were not detected in any of the sequencing-positive cases.

### FISH Screening for Fungal Infections

Fungal structures were detected in close association with tissue lesions in three cases, and fungal infection was diagnosed in all three cases. For two of these cases, fungal infection had been determined as the cause of abortion by routine methods (20). The FISH screening identified one additional case, which formerly was of unknown etiology. Based on the FISH result in combination with the histopathologic finding of necrosuppurative placentitis, this abortion case was reclassified as mycotic abortion.

### *Chlamydiaceae* and *Chlamydiales* PCR and Sequencing

All samples contained amplifiable DNA as verified by amplification of internal control targets. All 162 cases were PCR-negative for *Chlamydiaceae*. Nine cases were PCR-positive for *Chlamydiales*, and *Chlamydiales* DNA was detected only in the placenta of those cases. The Ct values for the positive cases ranged from 31.2 to 37.9 (Table 9). Amplicon sequences obtained from eight out of the nine positive cases allowed an assignment to the *Chlamydiales* order, thus confirming the qPCR results (Table 9). Specification on family or species level was not possible because of limited sequence length and quality.

**Table 9:** Sequencing results, histopathological findings, and etiologies for nine *Chlamydiales* PCR-positive bovine abortion cases.

**Case ID**	**Amplicon sequence assigned to *Chlamydiales*^a^**	**Ct value *Chlamydiales* PCR**	**Histopathological findings**	**Previous etiology**	**Final etiology**
Case 7	+	37.4	Necrosuppurative placentitis Suppurative bronchopneumonia Non-suppurative hepatitis Non-suppurative meningitis	Unknown	Unknown
Case 38	+	37.4	Suppurative placentitis Non-suppurative encephalitis	*S. aureus*	*S. aureus*
Case 45	+	32.1	Suppurative bronchopneumonia	Unknown	Unknown
Case 46	+	35.1	Suppurative bronchopneumonia	Unknown	Unknown
Case 47	–	36.7	No lesions (routine diagnostic group 4)	Unknown	Unknown
Case 48	+	37.4	Necrosuppurative placentitis Suppurative bronchopneumonia	Unknown	Unknown
Case 49	+	34.8	Suppurative placentitis	Unknown	Unknown
Case 50	+	31.4	Non-suppurative placentitis Non-suppurative encephalitis	Unknown	Unknown
Case 51	+	37.9	Suppurative bronchopneumonia Suppurative myocarditis	Unknown	Unknown

–*, no/negative; +, yes/positive.*

a *According to a BLAST search of the 16S ribosomal RNA (bacteria- and Archaea-type strains) database (https://blast.ncbi.nlm.nih.gov)*.

### *Leptospira* spp. qPCR

Using the 16S rRNA gene targeting assay, small amounts of *Leptospira* spp. DNA were detected in the liver (Ct = 35.6) and placenta (Ct = 39.1) from the only *Leptospira* 16S rDNA amplicon sequencing–positive case, whereas the kidney was negative. All three samples were negative when analyzed with the *lipL32* targeting assay.

### Diagnostic Rate, Final Etiology, and Most Sequencing-Abundant Bacterial Taxa

In total, the diagnostic rate for the 115 cases was increased from 46% (*n* = 53) to 53% (*n* = 61) (Table 7). This increase was the result of changing the etiologic diagnosis for 14 cases (12%) based on the combined methods approach. The detection of lesion association by FISH was the key aspect in changing diagnoses. In detail, the change of diagnoses was reached by the following: assigning a final etiology to 10 cases with a formerly unknown etiology [cases 2–4, 8, 16, 19, 20, 28, 33; Supplementary Tables 3, 5–7 and 1 mycotic abortion (data not shown)], changing the previously established etiology in two cases (cases 23, 44; Supplementary Table 6), and invalidating the previously established etiology due to the lack of pathogen–lesion association in two cases (cases 13, and 14; Supplementary Table 6). Furthermore, three cases formerly diagnosed with unknown or a single etiology were reclassified as dual infections (cases 3, 23, and 44; Table 6, Supplementary Table 6). The increase of diagnostic rate was not significant when tested for equal proportions (*P* = 0.356).

In order to evaluate how the most abundant bacterial taxa per case correlated with the cause of abortion, the final etiologic diagnosis was compared with the three most abundant taxa from the placenta and lung–liver pools for the 29 abortion cases, which had a final bacterial etiology (Table 8). On average, the final etiology was found among the three most abundant taxa of either placenta or lung–liver pool in 76% of the FISH screened cases. This was the highest accordance found. In 64% of the examined cases, the final etiology was among the three most abundant placental taxa, and in 61% of the cases, the final etiology was either the most abundant taxon in the placenta or the lung–liver pool.

## Discussion

The 16S rDNA amplicon sequencing approach was found to be a useful screening tool for potentially abortigenic bacterial infections due to its culture-independent high resolution of bacterial taxa present in bovine abortion samples.

The increase of the diagnostic rate for abortion cases with tissue lesions suggestive of infection was based on the hypothesis-free identification of potential bacterial abortifacients through deep sequencing and the subsequent verification of lesion association by FISH for a selection of the detected pathogens. The increase in diagnostic rate was insignificant when tested with an equal-proportions test. However, the numbers are small and without true-positives, it is difficult to meaningfully test the precision and sensitivity of the approach. We therefore reckon that the increased number of cases with a final etiologic diagnosis illustrates the diagnostic potential of the combined methods approach.

The cutoff value of 10% abundance (≥10,000 normalized reads/sample; 20% abundance for *E. coli*) was found to be a reasonable value for the selection of cases for the FISH screening, because the targeted species were detected in all screened cases, and it was possible to determine lesion association based on spatial distribution of the agent. For *C. burnetii* and *C. jejuni*, where all sequencing-positive cases were included in the FISH screening regardless of their abundance, the respective agent was not detected. For *C. burnetii*, this is probably due to the low abundance (≤ 1% in placenta). It might also be due to the presence of the respective species' 16S rDNA (target of the preamplification PCR) only and the lack of the less robust 16S rRNA (FISH target), or it might be due to a lower sensitivity of the respective FISH assay compared to the amplicon sequencing assay.

It has been recommended to supplement routine methods with molecular methods in abortion diagnostics in order to avoid underestimating the relevance of difficult-to-culture abortifacients like *C. burnetii, C. abortus*, and pathogenic *Leptospira* spp. (14). Assessing microbial communities based on rRNA genes relies upon true and accurate amplification of e.g., 16S rRNA genes from the original DNA samples (21). However, no diagnostic assay comes without a bias: the choice of primer sets as well as the number of amplification cycles influences how well the true composition of the bacterial community in a sample can be depicted. Primer 27f is one of the most commonly used primers for 16s rRNA genes (21, 39). The 27f primer binding sites of *C. burnetii* and *Chlamydiales* differ from most other known bacteria in one and three positions, respectively (21). These sequence differences are thought to hamper the detectability of members of these two taxa in 16S rDNA amplicon sequencing studies (39). In our study, we therefore chose a previously published 27f primer formulation that seeks to adjust for the amplification bias against *C. burnetii* and *Chlamydiales*. While *C. burnetii* was detected both alone and in the mixed positive control by 16S rDNA amplicon sequencing, *Chlamydia* was only retrieved from the positive controls spiked with *Chlamydia* DNA alone, but not from the mixed control containing *Chlamydia* spp., *C. burnetii*, and *L. interrogans* DNA. The *Chlamydiales*-adapted forward primer 27f-Chl amounted for only ca. 14% of the primer mixture, which might have favored the primer binding to the non-*Chlamydia* DNA during the first amplification cycles leading to an insufficient amplification of *Chlamydia* DNA below the sequencing assay's detection limit. In order to overcome the presumed amplification bias of our sequencing assay concerning *Chlamydia* and CLOs, we applied specific qPCR assays to screen for *Chlamydiaceae* and *Chlamydiales* DNA. In our study, the detected *Chlamydiales* were not found to be the cause of abortion based on the qPCR Ct values that were higher than those usually seen in clinically relevant infections. A study on *Parachlamydia* infections in deer considered Ct values between 35.0 and 38.0 as inconclusive (40). CLOs have been reported to play a role in bovine abortions in Switzerland and Scotland (41–43). However, contamination of placenta samples with environmental CLOs should be considered, because the majority of aborted placentas have environmental contamination from, e.g., feces and bedding. Contamination of bovine placentas with environmental *Parachlamydia* has been suggested based on 16S rDNA sequencing results from placenta and cattle drinking water (43).

While the liver and placenta of one abortion case were PCR-positive for *Leptospira* spp. with the 16S qPCR assay, all organ samples tested negative using the *lipL32* qPCR. The observed discrepancy of PCR results might result from the differing abundance of gene copies per *Leptospira* genome (two copies of the 16S rRNA gene vs. one copy of *lipL32* gene) and thereby might reflect the slightly higher sensitivity of the 16S targeting qPCR that has been reported previously (29). Due to the very low number of *L. interrogans* sequencing reads in the liver, and the low amount of *Leptospira* DNA detected by PCR, the case was not diagnosed as *Leptospira*-associated abortion, but remained of unknown etiology. Bovine *Leptospira* associated abortion has not been diagnosed in Denmark yet. The detection of the small amount of *Leptospira* DNA by sequencing in this case was confirmed by a sensitive qPCR assay, which suggests that our sequencing assay would be suitable to detect *Leptospira* spp. when present in numbers required to cause a clinical infection and abortion. This finding might furthermore suggest that our sequencing assay had a sensitivity similar to the applied 16S *Leptospira* PCR. However, further investigation would be needed to confirm this hypothesis.

In 16S rDNA amplicon sequencing studies, the choice of potential contamination control is crucial for the degree of accuracy with which the composition of the bacterial community within samples is represented by the resulting data set (44). PCR on low DNA templates, such as the fairly sterile fetal internal organs, may lead to spurious results, since minor contamination inherent in the reagents may dominate the sequences (45). On the other hand, e.g., some placenta samples were severely and diversely contaminated by often days of exposure to contaminated environment. Because of the heterogeneity of samples regarding bacterial load, the sequencing data from our study were mainly used to investigate known pathogens rather than for explorative purposes.

In one of the cases with unknown previous etiology (case 9, Supplementary Table 6), *T. pyogenes* was potentially missed to be identified as abortifacient: bacterial culture of fetal samples was negative for *T. pyogenes* and the placenta was sequencing-negative for *T. pyogenes*, i.e., the case did not match the inclusion criteria for the *T. pyogenes* FISH screening. However, *T. pyogenes* reads were detected in the respective extraction control as well as in the raw data (before subtraction of potential contaminant sequences shared with extraction controls and NTCs) of kidney, lung–liver pool, and placenta from this case. A retrospective FISH examination demonstrated the lesion association of *T. pyogenes* in the lung and placenta and suggested *T. pyogenes* as the most likely cause of abortion in this case (data not shown). This case, together with the *E. coli* cases sharing *E. coli* sequences with their extraction controls, illustrates the importance of a thorough evaluation of sequencing results together with all other diagnostic findings in abortion diagnostics. It also points out that balancing the stringency of potential contamination control in 16S rDNA amplicon sequencing studies is challenging and that false-negative results may be a consequence of the removal of potential contaminant sequences (44).

Our study showed that the combination of NGS with FISH has the potential to become a powerful tool for future abortion diagnostics due to the availability of rapid and inexpensive sequencing technologies and the availability of many specific pathogen-targeting probes. The combination of a hypothesis-free metataxonomic strategy with *in situ* pathogen detection has the potential to lead to more accurate diagnoses and to increase the diagnostic rate as it has been shown for other veterinary diagnostic fields (46).

## Conclusions

16S rDNA amplicon sequencing was found to be a useful screening tool for potentially abortigenic bacterial infections in cattle. The combination of routine methods with 16S rRNA amplicon sequencing and FISH increased the diagnostic rate for bovine abortion cases with lesions indicative of infection when compared to using routine diagnostic methods from 46 to 53%.

The pan-fungal FISH assay was well-suited for detecting fungal infections as cause of bovine abortions. The assay confirmed the minor importance of fungi as abortifacients in our study population.

Our study confirmed the assumption that *C. burnetii, Chlamydia* spp., and *Leptospira* spp. do not play a significant role as bovine abortifacients among the abortion cases submitted for diagnostics in Denmark.

## Data Availability Statement

The datasets presented in this study can be found in online repositories. The names of the repository/repositories and accession number(s) can be found at: https://www.ncbi.nlm.nih.gov/bioproject/PRJNA678972.

## Ethics Statement

Ethical review and approval was not required for the animal study because the study used bovine abortion material submitted for diagnostics. Written informed consent for participation was not obtained from the owners because the study used bovine abortion material submitted for diagnostics.

## Author Contributions

GWJ: conceptualization, methodology, investigation, and writing—original draft. MS: formal analysis. KS: conceptualization. CS: resources and supervision. TJ: conceptualization, funding acquisition, and project administration. JA: Writing—review and editing. All authors contributed to the article and approved the submitted version.

## Conflict of Interest

The authors declare that the research was conducted in the absence of any commercial or financial relationships that could be construed as a potential conflict of interest.
