# Supplementary material for: Bovine Abortions Revisited—Enhancing Abortion Diagnostics by 16S rDNA Amplicon Sequencing and Fluorescence in situ Hybridization
Source: Front Vet Sci. 2021 Feb 23;8:623666. doi: 10.3389/fvets.2021.623666 (PMC7940352; doi:10.3389/fvets.2021.623666)
Supplement: Supplementary file 1 [file Data_Sheet_1.pdf]

Supplementary Material

1 Material and methods

1.1 Animals

Fetal membranes and fetuses or fetal tissue from 153 bovine abortions and 9 stillbirths submitted to the National Veterinary Institute, Denmark, for diagnostics from January 2015 until June 2017 were examined. The material originated from 147 singleton and 15 twin pregnancies. The twin pregnancies were represented by 26 fetuses. The samples included in this study originated from a previous study (1) in which the etiology of abortion was investigated by routine diagnostic methods, i.e. macroscopic and histologic examination, aerobe bacterial culture (fetal lung, liver, abomasal content), and maternal serology for BVDV infection as well as BVDV immunohistochemistry in abortion cases from herds with a BVD-positive status. Each fetus or set of fetal organs counted as a single submission except for twin abortions with both twins submitted, which counted as one submission. Age determination of the fetus was based on insemination and abortion date, anamnestic data, and crown-rump-length (CRL) in decreasing priority (2). In cases lacking data on all of the aforementioned criteria, pulmonary histology was used to estimate fetal age (3). Abortions were categorized as having occurred during first, second or third trimester of gestation, i.e. gestation months ≤3, 4-6, and ≥7.

1.2 Reproduction data

Information on insemination and abortion dates, breed of the dam as well as number and gestational age of registered bovine abortions in Denmark was retrieved from the Danish Cattle Database (SEGES P/S, Aarhus, Denmark).

1.3 Necropsy and histopathological procedures

The fetuses and placentas were examined as described previously (1). Specimens for histology were collected from placenta, lung, liver, heart, kidney, and brain, fixed in 10% neutral buffered formalin, processed by routine methods, and embedded in paraffin. Tissue sections of 3-5 µm were cut and stained with hematoxylin and eosin (HE). Sections of placental specimens that were suspicious for fungal infection based on histological findings were additionally stained with Grocott's methenamine silver. For PCR and sequencing, specimens from placenta (up to three cotyledons), lung, liver, and

kidney were transferred into sterile plastic containers, frozen at -20 ˚C for up to 4 weeks and then stored at -80 ˚C until nucleic acid extraction. Lung and liver specimens were pooled, while kidney and placenta were analyzed separately.

1.4 Bacteriological procedures

Culturing was carried out as described previously (1). In brief: specimens of liver, lung, and abomasal contents were cultured under aerobic conditions at 37 ˚C on Columbia agar plates supplemented with 5% calf blood (in house) as well as on Drigalski lactose agar plates (in house). Selection of colonies for subculturing was based on colony morphology and quantity of those colony types that were not considered as post mortem contamination (e.g. Proteus spp.) based on the experience of the bacteriologist in charge. In case of mixed bacterial growth, bacteria from the three most numerous and significant colony types were subcultured. If more than three different colony types were present without any significant difference in their organ distribution, bacterial growth was described as an unspecific mixed flora and subculturing was not done. Colony material from the subcultures was analyzed by matrix-assisted laser desorption/ionization time-of-flight mass spectrometry (MALDI-TOF MS) as described previously (1). Lung, liver, and spleen were screened for *Brucella* spp. by culture on selective media (1).

1.5 Diagnostic criteria routine methods

Based on the diagnostic findings obtained with routine methods, the abortion cases were classified into four groups of diagnostics certainty that were described previously (1). In brief:

Group 1. Likely cause of abortion identified. Diagnosis of bacterial infection was based on isolation of bacteria and the presence of lesions consistent with bacterial infection. Mycosis was diagnosed by the presence of hyphae or yeast cells in tissue sections associated with corresponding inflammation. Protozoal abortion was diagnosed by findings of typical non-suppurative inflammation in fetal organs. Infection with BVDV was diagnosed by demonstration of BVDV antigen within fetal tissues.

Group 2. Lesions present, specific etiology not identified. This group included cases for which a specific cause was not detected but where lesions were present in the fetus and/or the fetal placenta. Cases were categorized based on the most severe lesion observed.

Group 3. Bacteria isolated from at least two organs without corresponding lesions. This group consisted of cases from which potentially pathogenic bacteria were isolated from at least two organs, but without associated lesions.

Group 4. No likely cause identified. This group comprised cases without fetal or placental lesions including cases with isolation of bacteria from only one organ, though without associated lesions.

1.6 Study population

During the study period, 20581 bovine abortions were registered in Denmark, whereof the abortion cases from our study comprised 0.8%. Most fetuses from our study (67%) were aborted during the last trimester, while 33% were aborted during the second trimester, and 1% during the first trimester. The distribution of gestational age of all registered abortions during the study period was: 3% first trimester, 20% second trimester, 77% last trimester.

2 Abbreviations

BVD	Bovine virus diarrhea
BVDV	Bovine viral diarrhea virus

3 Supplementary tables

Supplementary Table 1. Primer and probe sequences used for the detection of *Chlamydiaceae* and *Chlamydiales*.

Primer or probe name	Target	Sequence 5' - 3'	Reference
Ch23S-F	*Chlamydiaceae* 23S rRNA gene	CTGAAACCAGTAGCTTATAAGCGGT	(4)
Ch23S-R	*Chlamydiaceae* 23S rRNA gene	ACCTCGCCGTTTAACTTAACTCC	(4)
Ch23S-p	*Chlamydiaceae* 23S rRNA gene	FAM-CTCATCATGCAAAAGGCACGCCG-TAMRA	(4)
ACT2-1030-F	Bovine β-actin gene	AGCGCAAGTACTCCGTGTG	(5)
ACT-1135-R	Bovine β-actin gene	CGGACTCATCGTACTCCTGCTT	(5)
ACT-p-1081-YAK	Bovine β-actin gene	YAK-TCGCTGTCCACCTTCCAGCAGATGT-BHQ1	(5)
panCh16F2	*Chlamydiales* 16S rRNA gene	CCGCCAACACTGGGACT[a]	(6)
panCh16R2	*Chlamydiales* 16S rRNA gene	GGAGTTAGCCGGTGCTTCTTTAC[a]	(6)
panCh16S	*Chlamydiales* 16S rRNA gene	FAM-CTACGGGAGGCTGCAGTCGAGAATC-BHQ1[a]	(6)

BHQ1 = black hole quencher 1, FAM = 6-carboxyfluorescein, TAMRA = 5-carboxytetramethylrhodamin, YAK = Yakima Yellow

[a]The underlined nucleic acids are locked.

Supplementary Table 2. Control tissues used for the fluorescence *in situ* hybridization (FISH) screening of aborted tissues for selected bacterial species and fungi in general. All tissues were formalin-fixed and paraffin embedded.

**Control species (strain/type)**	**Tissue**	**Species**	**Origin**
C. abortus	placenta	ovine	spontaneous infection
C. burnetii	placenta	bovine	spontaneous infection
C. jejuni subsp. *jejuni* (ATCC 33560T)	lung	porcine	injected control[a]
C. pecorum	intestine	bovine	spontaneous infection
B. licheniformis	lung	murine	experimental infection
E. coli (serotype O138)	lung	porcine	injected control
E. coli (ATCC 25922)	liver	mouse	experimental infection
L. monocytogenes field isolate	liver	chinchilla	spontaneous infection
T. pyogenes field isolate	lung	porcine	injected control
Fungus with septate hyphae	placenta	cattle	spontaneous infection

[a]Pure bacterial cultures suspended in a 0.9% sterile saline solution were injected into sterile porcine lung samples. The tissue was then fixed in 10% neutral buffered formalin, processed routinely, embedded in paraffin, and cut into of 3-5 µm sections.

Supplementary Table 3. Diagnoses and results of bacterial culture, 16S rDNA amplicon sequencing, fluorescence *in situ* hybridization (FISH), and histology for bovine abortion cases that were FISH-screened for *Bacillus licheniformis* lesion association.

**Case ID**	**Culture *B. licheniformis* ≥1 organ**	**Sequencing >10,000 *B. licheni-formis* reads placenta**	**Sequencing *B. licheni-formis* in extraction control**	**Sequencing *B. licheni-formis* among 3 most abundant placenta**	**Sequencing *B. licheni-formis* among 3 most abundant lung-liver**	**Sequencing most abundant placenta**	**Sequencing most abundant lung-liver**	**FISH *B. licheni-formis* detected**	**FISH *B. licheni-formis* lesion-associ-ated**	**Histopathological findings**	**Previous etiology**	**Final etiology**
Case 1	+	+	-	+	-	*Fusobacterium periodonticum*	*Psychrobacter* unclassified	+	+	suppurative placentitis suppurative bronchopneumonia	*B. licheniformis*	*B. licheniformis*
Case 2	+	+	-	+	+	*Acinetobacter johnsonii*	*B. licheniformis*	+	+	necrosuppurative placentitis	unknown	*B. licheniformis*
Case 3	-	+	-	+	-	*B. licheniformis*	*Kurthia gibsonii*	+	+	necrosuppurative placentitis	unknown	*B. licheniformis, E. coli*
Case 4	-	+	-	+	-	*B. licheniformis*	*Shigella sonnei*	+	+	fibrinonecrotizing placentitis suppurative bronchopneumonia	unknown	*B. licheniformis*

− = no/negative; + = yes/positive.

Supplementary Table 4. Diagnoses and results of 16S rDNA amplicon sequencing, fluorescence *in situ* hybridization (FISH), and histology for bovine abortion cases that were FISH-screened for *Coxiella burnetii* lesion association.

**Case ID**	**Sequencing *C. burnetii* reads**	**Sequencing *C. burnetii* in extraction control**	**Sequencing *C. burnetii* among 3 most abundant placenta**	**Sequencing *C. burnetii* among 3 most abundant lung-liver**	**Sequencing most abundant placenta**	**Sequencing most abundant lung-liver**	**FISH *C. burnetii* detected**	**FISH *C. burnetii* lesion-associated**	**Histopathological findings**	**Previous etiology**	**Final etiology**
Case 5	+	-	-	-	*Clostridium sordellii*	*Vibrio vulnificus*	-	-	suppurative bronchopneumonia	*Klebsiella pneumoniae*	*Klebsiella pneumoniae*
Case 6	+	-	-	-	*T. pyogenes*	*T. pyogenes*	-	-	suppurative bronchopneumonia	*T. pyogenes*	*T. pyogenes*
Case 7	+	-	-	-	*Facklamia* unclassified	*Facklamia* unclassified	-	-	necrosuppurative placentitis<br>suppurative bronchopneumonia<br>non-suppurative hepatitis<br>non-suppurative meningitis	unknown	unknown

− = no/negative; + = yes/positive.

Supplementary Table 5. Diagnoses and results of 16S rDNA amplicon sequencing, fluorescence *in situ* hybridization (FISH), and histology for bovine abortion cases that were FISH-screened for *Campylobacter jejuni* lesion association.

**Case ID**	**Sequencing *C. jejuni***	**Sequencing *C. jejuni* among 3 most abundant placenta**	**Sequencing *C. jejuni* among 3 most abundant lung-liver**	**Sequencing *C. jejuni* in extraction control**	**Sequencing most abundnat at placenta**	**Sequencing highest reads lung-liver**	**FISH *C. jejuni* detected**	**FISH *C. jejuni* lesion-associated**	**Histopathological findings**	**Previous etiology**	**Final etiology**
Case 8	+	-	+	-	*E. coli*	*C. jejuni*	-	-	suppurative placentitis	unknown	*E. coli*

− = no/negative; + = yes/positive.

Supplementary Table 6. Diagnoses and results of bacterial culture, 16S rDNA amplicon sequencing, fluorescence *in situ* hybridization (FISH), and histology for bovine abortion cases that were FISH-screened for *Escherichia coli* lesion association.

**Case ID**	**Pure culture *E. coli* ≥1 organ**	**Sequencing >20,000 *E. coli* reads placenta**[a]	**Sequencing *E. coli* in extraction control**	**Sequencing *E. coli* among 3 most abundant placenta**	**Sequencing *E. coli* among 3 most abundant lung-liver**	**Sequencing most abundant placenta**	**Sequencing most abundant lung-liver**	**FISH *E. coli* detected**	**FISH *E. coli* lesion-associated**	**Histopathological findings**	**Previous etiology**	**Final etiology**
Case 3	+	-	-	-	-	*B. licheniformis*	*Kurthia gibsonii*	+	+	necrosuppurative placentitis	unknown	*B. licheniformis*, *E. coli*
Case 8	-	+	+	+	-	*E. coli*	*C. jejuni*	+	+	suppurative placentitis	unknown	*E. coli*
Case 9	-	+	-	+	-	*E. coli*	*Aerococcus viridans*	-	-	suppurative placentitis suppurative bronchopneumonia	unknown	unknown
Case 10	+	-	+	-	-	*Gemella*	*Gemella*	+	+	suppurative placentitis	non-hemolytic *E. coli*	*E. coli*
Case 11	+	-	-	-	-	*Streptococcus pluranimalium*	*Clostridium bifermentans*	+	-	non-suppurative necrotizing encephalitis non-suppurative myocarditis non-suppurative hepatitis	*N. caninum*	*N. caninum*

Case 12	+	-	-	-	-	*Streptococcus dysgalactiae*	*Clostridium sordellii*	+	+	suppurative placentitis<br>suppurative epicarditis	non-hemolytic *E. coli*	*E. coli*
Case 13	+	-	-	-	-	*Clostridium bifermentans*	*Romboutsia lituseburensis*	+	-	necrosuppurative placentitis<br>suppurative bronchopneumonia	non-hemolytic *E. coli*	unknown
Case 14	+	-	-	NA	-	NA	*Enterobacter hormaechei*	+	-	suppurative bronchopneumonia	non-hemolytic *E. coli*	unknown
Case 15	+	-	-	NA	-	NA	*Haemophilus influenzae*	-	-	suppurative bronchopneumonia<br>non-suppurative hepatitis<br>suppurative myocarditis	unknown	unknown
Case 16	-	+	-	+	+	*E. coli*	*E. coli*	+	+	necrosuppurative and fibrinous placentitis<br>suppurative bronchopneumonia	unknown	*E. coli*
Case 17	+	-	+	-	-	*Peptostreptococcus russellii*	*Clostridium bifermentans*	+	-	suppurative placentitis	unknown	unknown
Case 18	-	+	+	+	-	*E. coli*	*Proteus mirabilis*	-	-	suppurative bronchopneumonia<br>suppurative epicarditis<br>suppurative hepatitis	unknown	unknown

Case 19	-	+	-	+	+	*E. coli*	*Acinetobacter unclassified*	+	+	suppurative placentitis	unknown	*E. coli*
Case 20	-	+	-	+	-	*E. coli*	*Acinetobacter baumannii*	+	+	necrosuppurative placentitis<br>suppurative bronchopneumonia	unknown	*E. coli*
Case 21	-	+	-	+	-	*Clostridium perfringens*	*Acinetobacter baumannii*	+	-	suppurative placentitis<br>suppurative bronchopneumonia	unknown	unknown
Case 22	+	-	-	NA	-	NA	*Facklamia unclassified*	-	-	non-suppurative myocarditis<br>non-suppurative necrotizing encephalitis	*N. caninum*	*N. caninum*
Case 23	+	+	-	+	-	*E. coli*	*Acinetobacter unclassified*	+	+	necrotizing placentitis<br>non-suppurative myocarditis<br>non-suppurative hepatitis	*N. caninum*	*N. caninum, E. coli*
Case 24	-	+	+	+	-	*E. coli*	*Aerococcus viridans*	+	-	necrosuppurative placentitis<br>non-suppurative myocarditis<br>non-suppurative hepatitis<br>non-suppurative necrotizing encephalitis	*N. caninum*	*N. caninum*

Case 25	+	-	-	-	-	*Bacteroides unclassified*	*Clostridium butyricum*	+	-	suppurative placentitis	unknown	unknown
Case 26	-	+	-	+	-	*Streptococcus equinus*	*Fusobacterium necrophorum*	+	-	non-suppurative necrotizing placentitis<br>non-suppurative myocarditis<br>non-suppurative hepatitis<br>non-suppurative necrotizing encephalitis	*N. caninum*	*N. caninum*
Case 27	+	+	-	+	+	*Streptococcus pluranimalium*	*E. coli*	+	-	non-suppurative placentitis<br>non-suppurative myocarditis<br>non-suppurative hepatitis<br>non-suppurative necrotizing encephalitis<br>non-suppurative nephritis	*N. caninum*	*N. caninum*

− = no/negative; + = yes/positive.

[a]If placenta was not available, the number of reads from the lung-liver pool or lung were assessed.

Supplementary Table 7. Diagnoses and results of 16S rDNA amplicon sequencing, fluorescence *in situ* hybridization (FISH), and histology for bovine abortion cases that were FISH-screened for *Fusobacterium necrophorum* lesion association.

Case ID	Sequencing >10,000 *F. necrophorum* reads	Sequencing *F. necrophorum* in extraction control	Sequencing *F. necrophorum* among 3 most abundant placenta	Sequencing *F. necrophorum* among 3 most abundant lung-liver	Sequencing most abundant placenta	Sequencing most abundant lung-liver	FISH *F. necrophorum* detected	FISH *F. necrophorum* lesion-associated	Histopathological findings	Previous etiology	Final etiology
Case 12	+	-	+	-	*Streptococcus dysgalactiae*	*Clostridium sordellii*	+	-	suppurative placentitis<br>suppurative epicarditis	non-hemolytic *E. coli*	*E. coli*
Case 28	+	-	+	-	*Bacteroides* unclassified	*Lactococcus* unclassified	+	-	necrosuppurative placentitis<br>suppurative bronchopneumonia	unknown	*T. pyogenes*
Case 29	+	-	+	-	*F. necrophorum*	*Streptococcus equinus*	+	-	non-suppurative placentitis<br>non-suppurative myocarditis<br>non-suppurative necrotizing hepatitis	*N. caninum*	*N. caninum*

								non-suppurative encephalitis		
Case 30	+	-	+	-	*F. necrophorum*	*Lactococcus* unclassified	+	-	non-suppurative necrotizing placentitis non-suppurative myocarditis non-suppurative hepatitis non-suppurative necrotizing encephalitis	*N. caninum*	*N. caninum*
Case 31	+	-	+	-	*F. necrophorum*	*Lactococcus lactis*	+	-	suppurative placentitis	unknown	unknown
Case 32	+	-	+	-	*F. necrophorum*	*Staphylococcus equorum*	+	-	non-suppurative placentitis non-suppurative myocarditis non-suppurative necrotizing hepatitis non-suppurative necrotizing encephalitis	*N. caninum*	*N. caninum*
Case 33	+	-	+	-	*T. pyogenes*	*Enterococcus durans*	+	-	necrosuppurative placentitis suppurative bronchopneumonia	unknown	*T. pyogenes*

		non-suppurative encephalitis

− = no/negative; + = yes/positive.

Supplementary Table 8. Diagnoses and results of bacterial culture, 16S rDNA amplicon sequencing, fluorescence *in situ* hybridization (FISH), and histology for bovine abortion cases that were FISH-screened for *Listeria monocytogenes* lesion association.

**Case ID**	**Culture *L. monocytogenes* ≥1 organ**	**Sequencing >10,000 *L. monocytogenes* reads**	**Sequencing *L. monocytogenes* in extraction control**	**Sequencing *L. monocytogenes* among 3 most abundant placenta**	**Sequencing *L. monocytogenes* among 3 most abundant lung-liver**	**Sequencing most abundant placenta**	**Sequencing most abundant lung-liver**	**FISH *L. monocytogenes* detected**	**FISH *L. monocytogenes* lesion-associated**	**Histopathological findings**	**Previous etiology**	**Final etiology**
Case 34	+	+	-	+	+	*L. monocytogenes*	*L. monocytogenes*	+	+	suppurative placentitis non-suppurative encephalitis	*L. monocytogenes*	*L. monocytogenes*
Case 35	+	+	-	+	+	*L. monocytogenes*	*L. monocytogenes*	+	+	necrosuppurative placentitis	*L. monocytogenes*	*L. monocytogenes*

− = no/negative; + = yes/positive.

Supplementary Table 9. Diagnoses and results of bacterial culture, 16S rDNA amplicon sequencing, fluorescence *in situ* hybridization (FISH), and histology for bovine abortion cases that were FISH-screened for *Staphylococcus aureus* lesion association.

**Case ID**	**Culture *S. aureus* ≥1 organ**	**Sequencing >10,000 *S. aureus* reads**	**Sequencing *S. aureus* in extraction control**	**Sequencing *S. aureus* among 3 most abundant placenta**	**Sequencing *S. aureus* among 3 most abundant lung-liver**	**Sequencing most abundant placenta**	**Sequencing most abundant lung-liver**	**FISH *S. aureus* detected**	**FISH *S. aureus* lesion-associated**	**Histopathological findings**	**Routine etiology**	**Final etiology**
Case 36	+	+	-	+	-	*S. aureus*	*Psychrobacter psychrophilus*	+	+	necrotising placentitis<br>portal hepatitis	*S. aureus*	*S. aureus*
Case 37	+	-	-	-	-	*Psychrobacter psychrophilus*	*Staphylococcus equorum*	-	-	non-suppurative placentitis	unknown	unknown
Case 38	+	+	-	+	+	*S. aureus*	*S. aureus*	+	+	suppurative placentitis<br>non-suppurative encephalitis	*S. aureus*	*S. aureus*
Case 39	+	+	-	+	+	*S. aureus*	*S. aureus*	+	+	suppurative placentitis	*S. aureus*	*S. aureus*

− = no/negative; + = yes/positive.

4 References

1. Wolf-Jäckel GA, Hansen MS, Larsen G, Holm E, Agerholm JS, Jensen TK. Diagnostic studies of abortion in Danish cattle 2015-2017. *Acta Vet Scand* (2020) **62**:1. doi:10.1186/s13028-019-0499-4

2. University of Wisconsin. Bovine crown-rump-length calculator. Available at: http://www.ansci.wisc.edu/jjp1/ansci_repro/lab/lab12_03/crown-rump_analysis3.html [Accessed August 15, 2018]

3. Schnorr B. "Entwicklung der Atmungsorgane [Development of the respiratory system]," in *Embryologie der Haustiere* (Ferdinand Enke).

4. Ehricht R, Slickers P, Goellner S, Hotzel H, Sachse K. Optimized DNA microarray assay allows detection and genotyping of single PCR-amplifiable target copies. *Mol Cell Probes* (2006) **20**:60–63. doi:10.1016/j.mcp.2005.09.003

5. Wernike K, Hoffmann B, Kalthoff D, König P, Beer M. Development and validation of a triplex real-time PCR assay for the rapid detection and differentiation of wild-type and glycoprotein E-deleted vaccine strains of Bovine herpesvirus type 1. *J Virol Methods* (2011) **174**:77–84. doi:10.1016/j.jviromet.2011.03.028

6. Lienard J, Croxatto A, Aeby S, Jaton K, Posfay-barbe K, Gervaix A, Greub G. Development of a new *Chlamydiales*-specific real-time PCR and its application to respiratory clinical samples. (2011) **49**:2637–2642. doi:10.1128/JCM.00114-11
